# Plasticizing Effects of Polyamines in Protein-Based Films

**DOI:** 10.3390/ijms18051026

**Published:** 2017-05-10

**Authors:** Mohammed Sabbah, Prospero Di Pierro, C. Valeria L. Giosafatto, Marilena Esposito, Loredana Mariniello, Carlos Regalado-Gonzales, Raffaele Porta

**Affiliations:** 1 Department of Chemical Sciences, University of Naples “Federico II”, 80126 Naples, Italy; m.sabbah@najah.edu (M.S.); giosafat@unina.it (C.V.L.G.); marilena.esposito2@unina.it (M.E.); loredana.mariniello@unina.it (L.M.); raffaele.porta@unina.it (R.P.); 2 Department of Nutrition and Food Technology, An-Najah National University, P.O. Box: 7 Nablus, Palestine; 3 Departamento de Investigacion y Posgrado en Alimentos, Facultad de Quimica, Universidad Autonoma de Queretaro, 76010 Queretaro, Mexico; carlosr@uaq.mx

**Keywords:** polyamines, spermidine, spermine, edible film, bitter vetch, plasticizer

## Abstract

Zeta potential and nanoparticle size were determined on film forming solutions of native and heat-denatured proteins of bitter vetch as a function of pH and of different concentrations of the polyamines spermidine and spermine, both in the absence and presence of the plasticizer glycerol. Our results showed that both polyamines decreased the negative zeta potential of all samples under pH 8.0 as a consequence of their ionic interaction with proteins. At the same time, they enhanced the dimension of nanoparticles under pH 8.0 as a result of macromolecular aggregations. By using native protein solutions, handleable films were obtained only from samples containing either a minimum of 33 mM glycerol or 4 mM spermidine, or both compounds together at lower glycerol concentrations. However, 2 mM spermidine was sufficient to obtain handleable film by using heat-treated samples without glycerol. Conversely, brittle materials were obtained by spermine alone, thus indicating that only spermidine was able to act as an ionic plasticizer. Lastly, both polyamines, mainly spermine, were found able to act as “glycerol-like” plasticizers at concentrations higher than 5 mM under experimental conditions at which their amino groups are undissociated. Our findings open new perspectives in obtaining protein-based films by using aliphatic polycations as components.

## 1. Introduction

The interest in protein-based films and coatings has increased considerably over recent years due to their advantages with respect to the conventional petroleum derived materials and to other biodegradable materials made of polysaccharides and/or lipids [1,2]. Protein films generally exhibit better gas barrier and mechanical properties than polysaccharide films but both generally possess poor water vapor barrier characteristics as a consequence of their hydrophilic nature [3,4,5,6,7].

One of the main additives of all bio-based edible films is the plasticizer, which is generally a small molecule such as glycerol (GLY) or sorbitol. The plasticizer is able to improve film extensibility and flexibility by decreasing the attractive intermolecular forces and increasing both free volume and chain mobility [8]. Our recent studies demonstrated the effectiveness of the aliphatic diamine putrescine, as well as of the polyamine (PA) spermidine (SPD), as alternative plasticizers for pectin edible films [9]. The PAs SPD and spermine (SPM) are reported to be essential components of all living cells [10,11,12] because they are involved in cellular growth and proliferation [13,14], the differentiation of immune cells and the regulation of inflammatory reactions [15,16]. Normal levels of PAs are maintained not only by endogenous and intestinal microorganism biosynthesis, but also by their exogenous supply through the diet [13,17,18] which provides the largest amount. Moreover, since the biosynthesis of PAs was shown to decrease with age, their content in the diet seems to be important in maintaining the full functionality of the different tissues in the elderly [19]. On the other hand, the very low toxicity of PAs, attested by an acute oral toxicity of 0.6 g/kg in rats [20] and by an LD_50_ value higher than 2 g/kg in mice [21], indicated their possible addition to film forming solutions (FFSs) in obtaining safe edible biobased materials.

Therefore, attention was focused on the possible effects of both SPD and SPM not only on the functionality of the polysaccharide-based films but also as plasticizers of the protein-based films, in comparison with a well-known and largely used plasticizer such as GLY. To this aim, protein concentrate, obtained from bitter vetch (*Vicia ervilia*; BV) seeds and recently proposed as a promising source for both edible films and biodegradable containers [22,23,24], was used as raw material of protein-based film forming solution. BV is an ancient grain legume crop, originated in the Mediterranean region, which can be found today in many countries around the world. This annual Vicia genus shows several favorable features, such as having high yields and being a cheap protein source resistant to drought and insects. Not only for these reasons, but also because of its high nutritional value, capacity of nitrogen fixation and ability to grow in poor soils, BV is widely cultivated for forage [25].

Here we present data showing the specific ability of SPD to act as an effective cationic plasticizer for protein-based edible films, both in the absence and presence of GLY.

## 2. Results and Discussion

Since the ability of both diamine putrescine and triamine SPD to act as plasticizers of polysaccharide-based films has been recently demonstrated [9], we were stimulated to investigate the ability of the PAs to play the same role in protein-based films. Therefore, a protein mixture, extracted from BV seeds previously characterized for their chemical composition and capacity to prepare biodegradable films and containers, was selected as biomaterial source [24]. The obtained BV protein concentrate (BVPC) was always used either after its heat-treatment at 80 °C for 30 min, to obtain denatured proteins, or after its mild incubation at 25 °C to preserve native protein structures. First of all, the changes of zeta potential and Z-average of the nanoparticles occurring in the BVPC FFSs were investigated at different pH values and different SPD and SPM concentrations, both in the absence and presence of GLY.

**Figure 1:**
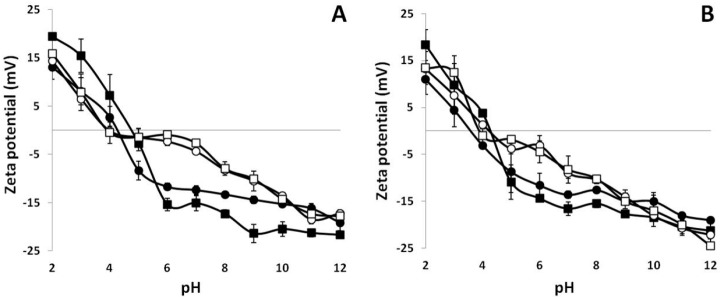
Effect of 5 mM spermidine (SPD) on zeta potential of 25 °C-treated (**A**) and 80 °C-treated (**B**) bitter vetch protein concentrate (BVPC) film forming solutions (FFSs) measured at different pH values in the absence or presence of 42 mM glycerol (GLY). The different FFSs contained only BVPC (●), BVPC + GLY (■), BVPC + SPD (○), BVPC + SPD + GLY (☐). The results are expressed as mean ± standard deviation. Further experimental details are given in the text.

**Figure 2:**
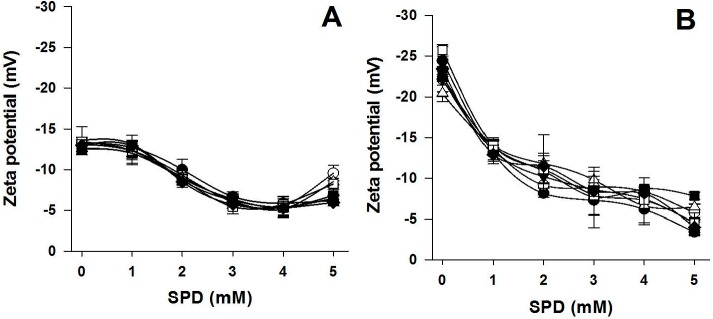
Effect of different SPD concentrations on 25 °C-treated (**A**) and 80 °C-treated (**B**) BVPC FFS zeta potential measured at pH 8.0 in the absence or presence of GLY. The different FFSs were prepared in the absence (●) or presence of 4 (○), 8 (▼), 17 (△), 25 (■), 33 (☐) and 42 mM (◆) GLY. The results are expressed as mean ± standard deviation. Further experimental details are given in the text.

As shown in Figure 1, high GLY concentrations (42 mM = 50% *w*/*w* protein) alone were found to significantly increase the negative zeta potential of all BVPC FFSs over pH 6.0 in the absence of PAs, probably as a consequence of the intermolecular hydrogen bonds formed by GLY with the electronegative atoms occurring in numerous reactive groups of BV proteins. Conversely, 5 mM SPD, present in BVPC FFSs during their heat-treatment both at 25 and 80 °C, always markedly decreased the negative zeta potential between pH 6.0 and 8.0, as a result of ionic interactions between the positively charged SPD amino groups and the BV negatively charged proteins in such pH range. Same results were obtained when 42 mM GLY was added to the FFSs after heat treatments, thus indicating that GLY did not significantly influence the SPD ionic interaction with BV proteins. As shown in Figure 2, the decreased negative zeta potential measured at pH 8.0 was dependent on SPD amount at all the GLY concentrations used and, as reported in Table 1, the SPM effect on zeta potential was even more pronounced than the SPD one, as well as not influenced by the presence of GLY.

Further experiments were carried out to determine the Z-average of nanoparticles occurring in the FFSs when BVPC was pretreated for 30 min either at 25 or 80 °C. The results reported in Table 2 and Table 3 indicate that, in both cases, the size of BV protein particles were always <1000 d.nm (and not significantly different) in the range of pH over 8.0, also in the presence of either SPD or SPM, their amino groups being uncharged and unable to ionically interact with BV proteins. Conversely, when PAs became positively charged, i.e., under pH 8.0, the protein mean particle size was found to increase over 1000 d.nm, as a result of PA/protein ionic bindings. Superimposable data were obtained both in the absence and presence of GLY. Therefore, these results confirm the ionic interaction of aliphatic PA amino groups with the negative charges occurring on the amino acid lateral chains of BV proteins under pH 8.0, as well as they indicate that the presence of GLY does not significantly influence PA/BV protein binding. In addition, when only GLY was present in the sample, a BV protein mean particle size >1000 d.nm was detected under pH 6.0, as a consequence of the formation of macromolecular aggregates in proximity of protein isoelectric points. In fact, GLY is a small molecule able to interact only with protein proton acceptors by intermolecular hydrogen bonds and BV aggregates were obtained under pH 6.0 also in the absence of GLY.

**Figure 3:**
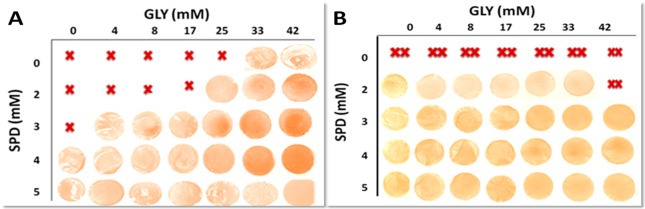
Films obtained by casting BVPC FFSs previously incubated for 30 min, either at 25 °C (**A**) or 80 °C (**B**), at pH 8.0 and in the presence of different concentrations of SPD and/or GLY. Not handleable–either brittle (X) or sticky (XX)-and handleable (photo) films are indicated. Further experimental details are given in the text.

**Figure 4:**
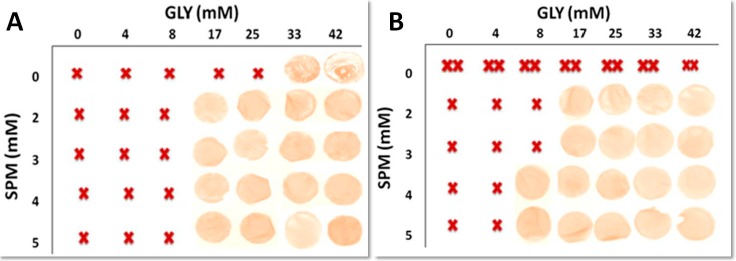
Films obtained by casting BVPC FFSs previously incubated for 30 min, either at 25 °C (**A**) or 80 °C (**B**), at pH 8.0 and in the presence of different concentrations of SPM and/or GLY. Not handleable –either brittle (X) or sticky (XX)- and handleable (photo) films are indicated. Further experimental details are given in the text.

Figure 3 and Figure 4 showed the results of the experiments carried out by casting the different FFSs prepared in the presence of either SPD or SPM, respectively, at pH 8.0 (i.e., when PAs became positively charged and the mean size of the particles occurring in the FFSs was <1000 d.nm) with the aim to determine the best conditions for obtaining handleable (i.e., not brittle nor sticky and, thus, easily to manipulate) and characterizable films. Panel A of both Figures shows that handleable yellowish films were formed in the presence of GLY alone at a minimal concentration of 33 mM (40%, *w*/*w* protein) by using not denatured BV proteins. Indeed, lower GLY concentrations gave rise to very brittle materials impossible to analyze unless SPD (Figure 3A), and less effectively SPM (Figure 4A) were present in the FFSs. But the most significant result was that handleable films were also obtained by adding SPD alone at a minimal concentration of 4 mM (Figure 3A), thus indicating that such PA is able to completely substitute GLY as a plasticizer not only to obtain polysaccharide–based [9] but also native protein-based films. Conversely, when denatured BV proteins were used, a different picture was recorded (Figure 3B), since quite sticky films were obtained by using GLY alone at all concentrations, whereas 2 mM SPD alone was sufficient to obtain handleable films both in the absence and presence of different GLY amounts. In addition, both panels of Figure 4 clearly indicate that SPM was absolutely unable to replace GLY as plasticizer by using both native and heat-denatured BV proteins, even though its presence reduces the amount of GLY needed to obtain handleable films.

In order to confirm the different interactions of SPD and GLY with BV proteins and investigate thermal behavior of the different films, differential scanning calorimetry (DSC) experiments were carried out. Preliminary DSC analyses indicated that both melting and glass transition temperature values of BV protein films prepared in the presence of 5 mM SPD were significantly higher (148 ± 2 °C and 70 ± 10 °C, respectively) than those measured with the films prepared in the presence of 42 mM GLY (130 ± 3 °C and 42 ± 7 °C, respectively), thus suggesting a more rigid structure determined by the ionic PA binding.

**Figure 5:**
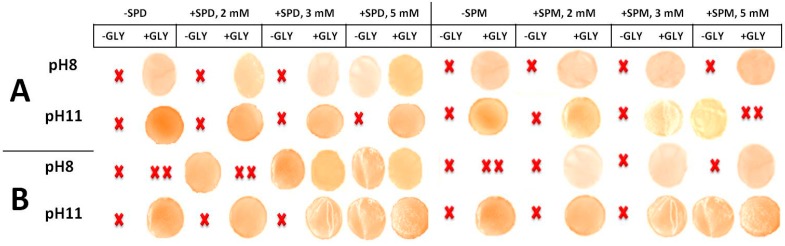
Films obtained by casting 25 °C-treated (**A**) and 80 °C-treated (**B**) BVPC FFSs previously incubated at either pH 8.0 or 11.0 in the presence of different concentrations of either SPD or SPM and/or 42 mM GLY. Brittle (X), sticky (XX) or handleable (photos) films are indicated. Further experimental details are given in the text.

Finally, Figure 5 compares native and denatured BV protein films obtained at pH 8.0 and pH 11.0 with different amounts of either SPD or SPM and in the absence or presence of a high concentration of GLY (42 mM). Figure 5B indicates that GLY allowed the formation of handleable films only at pH 11.0, by using heat-denatured BV proteins both in the absence and presence of PAs at all concentrations used. Conversely, the showed results indicate that GLY always allows to produce handleable films at both pH values, in the absence or presence of PAs, when native BV proteins were used, with the only exception of the sample containing 5 mM SPM which, at pH 11.0, gave rise to a very sticky material.

**Figure 6:**
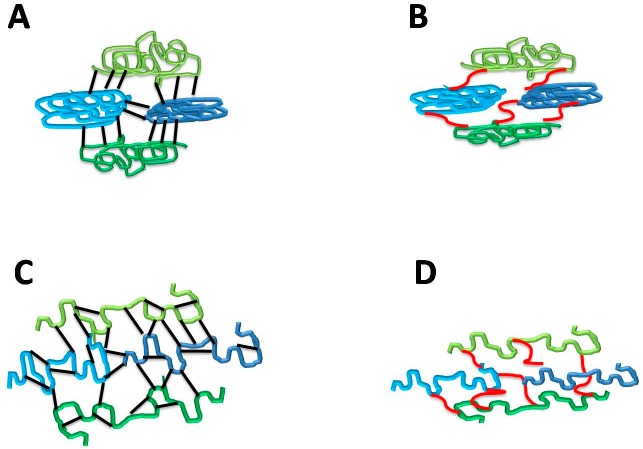
Proposed model of hydrogen bond GLY (black bar in **A**,**C**) and ionic SPD (red bar in **B**,**D**) interactions with BV proteins at pH 8.0 following FFS treatment for 30 min at either 25 °C (**A**,**B**, native proteins) or 80 °C (**C**,**D**, denatured proteins). The different kind of binding of GLY (hydrogen) and SPD (ionic) to BV proteins is proposed to be responsible for the different film thickness (minor thickness = major compactness for PA-containing film).

On the other hand, whereas lower concentrations of each PA alone resulted in the inability to produce handleable films at pH 11.0, as expected being uncharged over pH 8.0, surprisingly 5 mM of both PAs (SPD with only heat-denatured proteins) produced handleable films at pH 11.0 also in the absence of GLY (Figure 5A,B). These findings could be explained with the formation of hydrogen bonds between BV proteins and PA undissociated –NH_2_ groups, in a similar way to those formed by GLY –OH groups. Following this hypothesis both PAs, mainly SPM, could be considered also as “GLY-like plasticizers” when used at high concentrations (>5 mM) and at pH values that do not allow the dissociation of their amino groups. This assumption seems to be confirmed by the measured values of the thickness of films prepared at pH 11.0 in the presence of 5 mM PAs (83.60 ± 1.63 µm for SPD and 92.83 ± 2.31 µm for SPM containing heat-denatured protein films, respectively; 94.33 ± 2.54 µm for SPM containing native protein film) which were in the same order of magnitude as those detected for the GLY-containing films (95.10 ± 2.23 µm and 85.50 ± 2.08 µm for native and heat-denatured protein films, respectively) at pH 11.0. In contrast, as shown in Table 4, the measured thickness values of SPD-containing films prepared at pH 8.0 were always significantly lower in the absence of GLY, increasing with the increase of the concentration of GLY, thus suggesting that the ionic interaction of SPD with BV proteins affected a more compact film network, as compared to the one originating from the GLY-induced hydrogen bonds. A possible model of such a hypothesis is illustrated in Figure 6.

## 3. Materials and Methods

### 3.1. Materials

Bitter vetch seeds were purchased from a local market in Gallicchio (PZ), Italy. GLY (about 87%) was supplied from the Merck Chemical Company (Darmstadt, Germany), whereas SPD and SPM were from Sigma Chemical Company (St. Louis, MO, USA). All other chemicals and solvents used in this study were analytical grade commercial products.

### 3.2. BV Protein Concentrate Preparation

Proteins contained in BV seeds were extracted as previously described [22] with some modifications. The seeds were grinded by using a rotary mill (Grindomix GM200, Retsch GmbH, Haan, Germany) at speed of 1300 r.p.m. for 5 min and the flour was dispersed in distilled water (1:10, *w*/*v*), brought at pH 11.0 with 0.1 N NaOH and stirred at medium speed for 1 h at room temperature. After centrifugation at 3200× *g* for 10 min, the supernatant was collected and the pH adjusted at 5.4 by 0.1 N HCl addition to form a precipitate which was then separated by a new centrifugation at 3200× *g* for 10 min. Finally, the pellet was poured and uniformly distributed on a plastic plate and dried at 37 °C and 25% relative humidity. The obtained BVPC was finally grinded and its protein content (77%) determined by the Kjeldahl’s method [26], using a nitrogen conversion factor of 6.25.

To prepare the different FFSs, BVPC was dispersed in distilled water (2 g/100 mL) and the pH value was adjusted to pH 12.0 by using NaOH 0.1 N under constant stirring until the powder was completely solubilized. Aliquots of BVPC solution were then added with different concentrations of PAs and incubated for 30 min either at 25 or 80 °C to obtain FFSs containing both native and denatured BV protein samples. Two additional aliquots of BVPC solution were brought at pH 8.0 and 11.0, respectively, by 0.1 N HCl and then incubated in the presence of PAs for 30 min either at 25 or 80 °C. Where indicated, different concentrations of GLY were added to the obtained FFSs at the end of incubation.

### 3.3. FFS Zeta Potential and Mean Hydrodynamic Diameter Determination

Zeta potential and mean hydrodynamic diameter (Z-average) of the BVPC FFSs prepared at pH 12.0, containing or not high concentrations of PAs and/or GLY, were titrated automatically from pH 12.0 to pH 2.0 as previously described [5], by measuring the dynamic light scattering with a Zetasizer Nano-ZSP (Malvern^®^, Worcestershire, UK) using a He–Ne laser (wavelength of 633 nm) and a detector angle of 173°. The effect of different concentrations of PAs and GLY on both zeta potential and Z-average of FFSs were also studied with BVPC FFSs at pH 8.0 and 11.0 immediately after their preparation to prevent possible alterations in molecular interaction during storage [27]. 

### 3.4. BVPC Film Preparation

The same volumes (50 mL) of all the different FFSs, containing or not PAs and/or GLY, were poured onto 8 cm diameter polystyrene Petri dishes (7.8 mg BV proteins/cm^2^) and finally allowed to dry in an environmental chamber at 25 °C and 45% RH for 48 h. The handleable dried films were peeled intact from the casting surface and their thickness was immediately afterwards measured with a micrometer (Electronic digital micrometer, DC-516, sensitivity 0.001 mm) at different positions for each film sample to prevent possible film structural changes [27].

### 3.5. Differential Scanning Calorimetry Analysis

Thermal features of BV protein films were investigated by DSC (Perkin Elmer DSC-7) in which film samples of 6.0 mg were equilibrated at 25 °C for 2 min and heated to 240 °C at a rate of 20 °C/min.

### 3.6. Statistical Analysis

The data were subjected to statistical analyses by JMP software 5.0 (SAS Institute, Cary, NC, USA) and the means were compared using the Tukey-Kramer HSD test. Differences were considered to be significant at *p* < 0.05.

## 4. Conclusions

Zeta-potential and Z-average characterization of native and heat-denatured bitter vetch protein particles occurring in film forming solutions in the presence of the polyamines spermidine and spermine demonstrated that spermidine is able, specifically, to act as a cationic plasticizer in obtaining protein-based films. Conversely, spermine was found to only lower the minimal amount of glycerol needed to act as plasticizer by interacting with proteins through hydrogen bonds. All the spermidine containing films exhibited a significantly reduced thickness compared to the glycerol-plasticized ones, suggesting the formation of more compact protein networks due to ionic interactions between the positively charged polyamine and the protein negative charges. In addition, both polyamines, mainly spermine, showed to be able to act as “glycerol-like” plasticizers when their amino groups are undissociated.

The present findings open new perspectives in the preparation of a variety of protein-based edible films by using polyamines as plasticizers.

## Figures and Tables

**Table 1:** Effect of different concentrations of polyamines (PAs) on 25 °C-treated (**A**) or 80 °C-treated (**B**) BVPC FFSs zeta potential measured at either pH 8.0 or 11.0 in the absence and presence of 42 mM GLY *.

Addition		Zeta Potential (mV)			
		pH 8.0		pH 11.0	
		−GLY	+GLY	−GLY	+GLY
**A**	none	−13.30 ± 0.68	−13.00 ± 0.69	−23.5 ± 1.01	−20.5 ± 1.56
	+2 mM SPD	−10.00 ± 1.25 §	−8.48 ± 0.45 §	−22.2 ± 1.79	−21.8 ± 0.52
	+3 mM SPD	−6.68 ± 0.55 §	−5.26 ± 0.86 §	−23.5 ± 2.42	−23.6 ± 1.34
	+5 mM SPD	−3.92 ± 0.41 §	−3.57 ± 0.64 §	−22.1 ± 0.85	−22.3 ± 1.22
	+2 mM SPM	−5.09 ± 1.16 §	−6.13 ± 0.27 §	−21.1 ± 1.83	−22.9 ± 2.76
	+3 mM SPM	−3.40 ± 0.77 §	−4.00 ± 0.60 §	−23.6 ± 0.84	−24.1 ± 2.97
	+5 mM SPM	−1.80 ± 0.81 §	−2.20 ± 0.92 §	−20.5 ± 1.20	−21.4 ± 1.70
**B**	none	−14.40 ± 1.72	−13.40 ± 1.21	−22.0 ± 1.92	−21.9 ± 1.08
	+2 mM SPD	−8.13 ± 0.21 §	−11.50 ± 3.86	−24.9 ± 3.07	−25.9 ± 3.93
	+3 mM SPD	−7.27 ± 1.83 §	−8.42 ± 0.65 §	−25.6 ± 2.98	−24.5 ± 1.40
	+5 mM SPD	−3.40 ± 0.35 §	−4.01 ± 0.93 §	−21.4 ± 2.40	−22.2 ± 2.01
	+2 mM SPM	−4.69 ± 1.09 §	−7.35 ± 0.72 §	−22.7 ± 2.29	−26.8 ± 0.70
	+3 mM SPM	−2.34 ± 0.61 §	−2.43 ± 0.74 §	−21.6 ± 0.77	−21.2 ± 2.22
	+5 mM SPM	−1.83 ± 0.49 §	−1.62 ± 0.65 §	−20.1 ± 1.50	−20.7 ± 2.40

* The results are expressed as mean ± standard deviation. § Significantly different values as compared to the ones obtained under the same experimental conditions in the absence of PAs. Further experimental details are given in the text.

**Table 2:** Effect of 5 mM polyamines (PAs) on Z-average of the nanoparticles contained in the 25 °C-treated BVPC FFS measured at different pH values both in the absence and presence of 42 mM GLY *.

pH	Z-Average (d.nm)					
	None	+GLY	+SPD	+SPD/GLY	+SPM	+SPM/GLY
12	254.3 ± 14.4	272.5 ± 21.7	273.8 ± 2.0	280.4 ± 4.3	262.9 ± 9.3	258.5 ± 13.1
11	250.1 ± 13.6	283.9 ± 16.2	272.7 ± 14.0	282.3 ± 17.7	263.0 ± 2.3	346.8 ± 19.2
10	237.5 ± 14.0	270.4 ± 6.3	298.3 ± 12.0	312.7 ± 12.5	243.0 ± 4.8	336.1 ± 6.9
9	310.1 ± 23.8	292.5 ± 5.7	319.9 ± 11.4	471.6 ± 65.2	305.7 ± 38.6	423.4 ± 44.8
8	304.3 ± 44.0	289.4 ± 9.6	477.3 ± 100.4	424.9 ± 55.3	501.8 ± 5.6	816.7 ± 59.5
7	344.2 ± 30.2	358.0 ± 8.4	>1000 §	>1000 §	>1000 §	>1000 §
6	330.5 ± 16.3	335.7 ± 43.2	>1000 §	>1000 §	>1000 §	>1000 §
5	>1000 §	>1000 §	>1000 §	>1000 §	>1000 §	>1000 §
4	>1000 §	>1000 §	>1000 §	>1000 §	>1000 §	>1000 §
3	>1000 §	>1000 §	>1000 §	>1000 §	>1000 §	>1000 §

* The results are expressed as mean ± standard deviation. § Significantly different values as compared to the ones obtained under the same experimental conditions at higher pH. Further experimental details are given in the text.

**Table 3:** Effect of 5 mM polyamines (PAs) on Z-average of the nanoparticles contained in the 80 °C-treated BVPC FFS measured at different pH values both in the absence and presence of 42 mM GLY *.

pH	Z-Average (d.nm)					
	None	+GLY	+SPD	+SPD/GLY	+SPM	+SPM/GLY
12	228.9 ± 6.8	229.9 ± 5.4	242.5 ± 9.0	227.7 ± 5.4	320.0 ± 10.6	303.3 ± 35.5
11	234.5 ± 6.2	232.9 ± 1.3	229.6 ± 3.0	233.4 ± 3.0	370.8 ± 66.4	426.3 ± 21.6
10	225.4 ± 10.0	213.2 ± 3.3	210.6 ± 6.1	217.8 ± 3.9	329.0 ± 7.6	394.6 ± 77.2
9	206.6 ± 1.7	197.0 ± 1.4	187.2 ± 6.3	189.6 ± 10.2	370.7 ± 4.9	374.6 ± 17.0
8	187.9 ± 0.4	188.8 ± 1.3	227.7 ± 7.0	173.4 ± 3.6	560.6 ± 11.3	680.9 ± 90.0
7	195.4 ± 0.7	187.7 ± 8.7	>1000 §	>1000 §	>1000 §	>1000 §
6	179.4 ± 0.9	185.4 ± 6.0	>1000 §	>1000 §	>1000 §	>1000 §
5	>1000 §	>1000 §	>1000 §	>1000 §	>1000 §	>1000 §
4	>1000 §	>1000 §	>1000 §	>1000 §	>1000 §	>1000 §
3	>1000 §	>1000 §	>1000 §	>1000 §	>1000 §	>1000 §

* The results are expressed as mean ± standard deviation. § Significantly different values as compared to the ones obtained under the same experimental conditions at higher pH. Further experimental details are given in the text.

**Table 4:** Thickness (µm) of films obtained by casting 25°C-treated (**A**) and 80°C-treated (**B**) BVPC FFSs previously incubated at pH 8.0 in the presence of different concentrations of SPD and/or GLY *.

Components				A	B
GLY,	42 mM			100.20 ± 1.44	74.66 ± 1.50
SPD,	2 mM			58.12 ± 2.50 §	56.16 ± 2.87 §
	3 mM			59.43 ± 1.81 §	57.66 ± 1.63 §
	4 mM			67.66 ± 1.93 §	59.85 ± 2.51 §
	5 mM			79.75 ± 2.68 §	61.33 ± 1.75 §
GLY,	25 mM +	SPD,	2 mM +	79.50 ± 3.56 §	74.33 ± 2.65
GLY,	33 mM			87.83 ± 3.25 §	76.66 ± 2.25
GLY,	4 mM +	SPD,	3 mM +	57.50 ± 2.08 §	56.16 ± 1.72 §
GLY,	33 mM			105.67 ± 2.84	75.83 ± 3.06

* The results are expressed as mean ± standard deviation. § Significantly different values as compared to the ones obtained under the same experimental conditions in the presence of 42 mM GLY. Further experimental details are given in the text.
